# Inhaled corticosteroids for asthma: impact of practice level device switching on asthma control

**DOI:** 10.1186/1471-2466-9-1

**Published:** 2009-01-02

**Authors:** Mike Thomas, David Price, Henry Chrystyn, Andrew Lloyd, Angela E Williams, Julie von Ziegenweidt

**Affiliations:** 1 Department of General Practice and Primary Care, University of Aberdeen, Foresterhill Health Centre, Westburn Road, Aberdeen, UK; 2 Division of Pharmacy and Pharmaceutical Sciences, School of Applied Sciences, University of Huddersfield, Huddersfield, HD1 3DH, UK; 3 Oxford Outcomes Ltd, Oxford, UK; 4 Global Health Outcomes, GlaxoSmithKline R&D, Uxbridge, Middlesex, UK; 5 Respiratory Research, Ltd, Sankence, UK

## Abstract

**Background:**

As more inhaled corticosteroid (ICS) devices become available, there may be pressure for health-care providers to switch patients with asthma to cheaper inhaler devices. Our objective was to evaluate impact on asthma control of inhaler device switching without an accompanying consultation in general practice.

**Methods:**

This 2-year retrospective matched cohort study used the UK General Practice Research Database to identify practices where ICS devices were changed without a consultation for ≥5 patients within 3 months. Patients 6–65 years of age from these practices whose ICS device was switched were individually matched with patients using the same ICS device who were not switched. Asthma control over 12 months after the switch was assessed using a composite measure including short-acting β-agonist and oral corticosteroid use, hospitalizations, and subsequent changes to therapy.

**Results:**

A total of 824 patients from 55 practices had a device switch and could be matched. Over half (53%) of device switches were from dry powder to metered-dose inhalers. Fewer patients in switched than matched cohort experienced successful treatment based on the composite measure (20% vs. 34%) and more experienced unsuccessful treatment (51% vs. 38%). After adjusting for possible baseline confounding factors, the odds ratio for treatment success in the switched cohort compared with controls was 0.29 (95% confidence interval [CI], 0.19 to 0.44; p < 0.001) and for unsuccessful treatment was 1.92 (95% CI, 1.47 to 2.56; p < 0.001).

**Conclusion:**

Switching ICS devices without a consultation was associated with worsened asthma control and is therefore inadvisable.

## Background

Much of the costs of asthma are associated with suboptimally controlled disease [1-6]. By one estimate, based on an analysis of several studies, one third of the direct medical costs and three quarters of the total costs of asthma can be attributed to uncontrolled disease [2]. For patients with persistent asthma, inhaled corticosteroids (ICS) are considered to be the most effective controller medications currently available [7]. A range of different molecules and inhaler devices are marketed, and more are regularly being developed, often resulting in several options for ICS delivery.

Each type of inhaler device has its advantages and disadvantages together with cost [8], and each requires a different inhalation technique. In a recent review of inhaler devices by Virchow et al (2008), device characteristics were compared. Virchow et al suggested that the choice of inhaler may be one reason why asthma remains poorly controlled [9]. Patient training for each device and regular checking of inhalation technique are, therefore, considered to be essential for optimal treatment delivery [8,10-12]. In fact, many believe, and asthma management guidelines reinforce, that an inhaler should not be prescribed without confirming that the patient knows how to use the device [11,13].

Prescription cost-containment measures are increasing in many countries and, as more devices become available, there may be pressure to switch patients to a cheaper inhaler device [14]. Indeed, in some countries, such a substitution is mandated by current regulations, and patients who do not accept the substitution have to pay the difference in cost. However, acquisition cost savings from a substitution could be offset by costs related to deterioration in asthma control if the patient is unable or unwilling to use the inhaler device properly.

The purpose of this retrospective database analysis was to evaluate the impact on asthma control of inhaler device switching without an accompanying consultation in general practice in the United Kingdom (UK). Our hypothesis was that, for patients with asthma, switching inhaler device without a consultation is associated with worsened clinical outcomes.

## Methods

### Study design

This 2-year retrospective matched cohort study included a baseline period of 1 year before the date of ICS device switch (index date) and an outcome period of 1 year after the index date. Patient data were drawn from 1990–2004 records in the General Practice Research Database (GPRD), a large computerized database of anonymized longitudinal medical records from primary care in the Uk [15]. The years of the switch and matched cohort analyzed in this study are 1992 to 2003. This database includes information for over 13 million patients, including 3.4 million active patients for whom data are currently being gathered. Reports for each patient include demographic data and event history, including date and type of event with a description of the event and the corresponding code. Information about the reason for a switch in device is not recorded. Ethical approval was received from the GPRD Scientific and Ethical Advisory Committee (Protocol 706R, 26.4.05).

### Patients

Patients eligible for inclusion in this analysis were receiving ICS, were 6–65 years of age on the index date, and had a recorded diagnosis of asthma and no record of chronic obstructive pulmonary disease (COPD). We used the GPRD to identify primary care practices where ICS inhaler devices were switched without an accompanying consultation on the same day for five or more patients within a 3-month period. Patients had to be registered at the practice for at least 1 year before and 1 year after the index date. We included switches to inhaler devices that require training to use, namely, from a dry powder inhaler to a metered-dose inhaler, another brand of dry powder inhaler, or a breath-actuated inhaler or from a breath-actuated to a metered-dose inhaler or another brand of breath-actuated inhaler. Patients changed to a device recorded as 'generic' were excluded.

Each patient included in the analysis was individually matched to another patient (from any practice) who was receiving the same ICS device on the index date and met the following six criteria: same sex, age within 5 years, same smoking status, match on presence or absence of rhinitis history, and, during the prior year, same number of oral corticosteroid prescriptions and hospitalizations for asthma.

### Outcome measures

Outcome measures were analyzed for the year before (baseline period or year 1) and the year after the index date (outcome period or year 2). The primary outcome measure was a composite measure of asthma control, as defined in Table 1. Secondary outcome measures included several individual elements of asthma-related health resource utilization. The number of prescriptions/patient per year was analyzed for inhaled short-acting β-agonist and oral corticosteroids; in addition, the number of doses/day of short-acting β-agonist was determined for each patient based on the number of inhalers prescribed/year and an assumed standard dose of salbutamol 200 μg and terbutaline 500 μg. We also analyzed the number of general practice consultations (physician and practice nurse) for asthma, the number of hospital admissions for asthma, and the number of hospital admissions for possible asthma, which were defined as nonspecific hospitalization code and asthma-related code within a 1-week window.

**Table 1:** Definition of asthma control – a composite measure – over 12 months

	Assessment of Asthma Control*		
	
Criterion for the Composite Measure	Successful Treatment (All Criteria)	Partially Successful Treatment (All Criteria)	Unsuccessful Treatment (≥ 1 Criterion)
Mean SABA dose	≤0.5 dose/day	0.5 to ≤2 doses/day	>2 doses/day
Oral corticosteroid use/yr	None	≤2 courses	≥3 courses
Controller therapy	Not changed	Not changed	Changed
Hospitalization†	None	None	≥1

SABA = short-acting β-agonist; 1 daily dose = salbutamol 200 μg or terbutaline 500 μg.

*Successful and partially successful treatment had to meet each criterion, while unsuccessful treatment could be defined by any one or more of the criteria.

†This includes any hospital attendance for asthma, including admission, emergency department, or outpatient department (hospital clinic) attendance.

Other data recorded for each patient included assigned socioeconomic status of the primary care practice and other respiratory or confounding diagnoses. The daily dose of ICS was calculated from the prescribing instructions on the first available prescription after the index date.

### Statistical analyses

Baseline characteristics of switched and matched control patients were compared using *t *and χ^2 ^tests as appropriate. Median intended ICS dose, daily short-acting β-agonist dose, and asthma consultation pattern during the baseline year were compared for the two cohorts using the Mann-Whitney *U *test. For comparison of change between year 1 and year 2, the paired *t *test (mean changes in short-acting β-agonist use and asthma consultations) and χ^2 ^test (change or not in ICS dose or device from year 1 to year 2) were used.

For comparisons of the primary outcome measure, in addition to the unadjusted analyses, we used logistic regression to correct for potential baseline confounders. Binary regression models were constructed using as the three outcome variables 1) successful vs. partially successful/unsuccessful treatment, 2) unsuccessful treatment vs. successful/partially successful treatment, and 3) subsequent change in ICS device or dose and, as explanatory variables, cohort membership plus possible confounding factors. Possible baseline confounders entered into the model included 1) short-acting β-agonist use (average daily use categorized as none, ≤0.5 doses/day, >0.5–2 doses/day, >2 doses/day); 2) asthma consultation rate (categorized as: none, 1, 2, ≥3); 3) socioeconomic status (in quintiles); and 4) prescribed ICS dose at switch (in sensitivity analysis as incomplete data due to some patients receiving vague prescribing instructions, categorized as ≤400 μg/day, 401–800 μg/day, 801–2000 μg/day, >2000 μg/day).

## Results

### Patients

**Figure 1:**
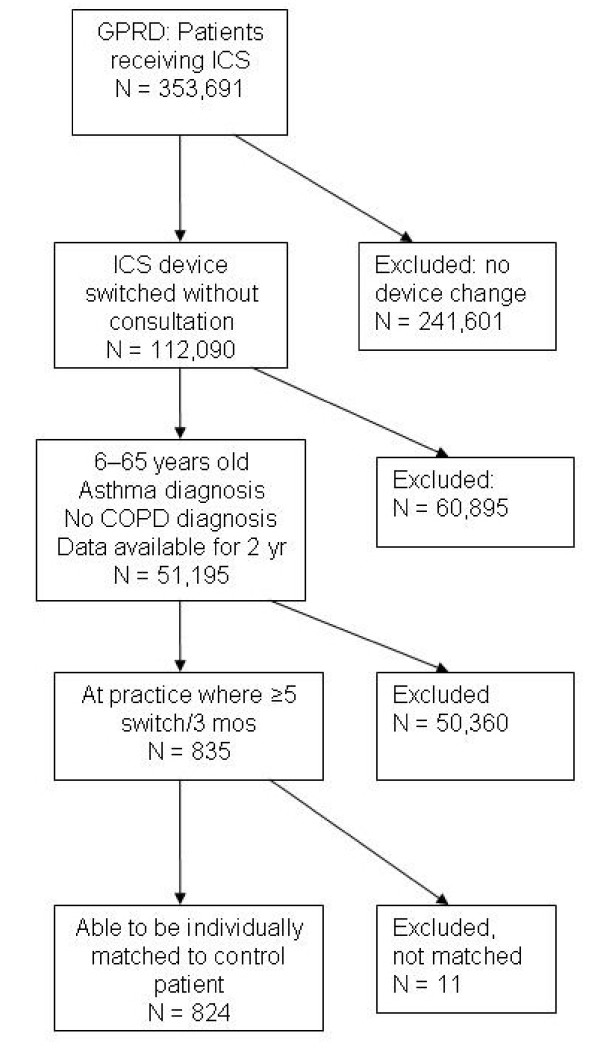
**Study flow diagram showing patient selection for the switched cohort**.

We identified 112,090 patients with asthma in the GPRD database who had a switch of ICS device (Figure 1). There were 55 primary care practices in the database where a switch in device without an accompanying consultation occurred on five or more occasions over 3 months. Overall, at these 55 practices, 835 patients had a device switch, and 824 could be matched according to our criteria. The number of switches ranged from 5 to 127 per practice; ≥10 patients were switched at 24 practices, and ≥30 patients were switched at 7 practices. Over half (53%) of the device switches were from a dry powder to a metered-dose inhaler (Table 2).

**Table 2:** Types of inhaled corticosteroid (ICS) device switch for 824 patients whose device was switched without an accompanying clinical visit or consultation

Type of ICS device switch	No. (%) of patients (n = 824)
Dry powder to metered-dose inhaler	437 (53)
Dry powder to dry powder inhaler (different brand)	106 (13)
Dry powder to breath-actuated inhaler	151 (18)
Breath-actuated to metered-dose inhaler	105 (13)
Breath-actuated to breath-actuated inhaler (different brand)	25 (3)

The switched and control cohorts were identical or comparable, as per the study protocol, with regard to the criteria used for matching (Table 3). The rate of admissions associated with a respiratory consultation was also similar at baseline in the switched and control cohorts (5 vs. 9 patients with 6 vs. 10 admissions, respectively), as was the presence of recorded gastroesophageal reflux or cardiovascular disease (see Table 3).

**Table 3:** Baseline and demographic characteristics of the two study cohorts: patients whose inhaled corticosteroid (ICS) device was switched (switched cohort) and the matched patients whose ICS device was not switched (control cohort)

Baseline Characteristic		Switched Cohort (n = 824)	Control Cohort (n = 824)	p Value For Comparison
Sex*	Female, n (%)	445 (54%)	445 (54%)	N/A
Age* in yrs	Median (IQR)	32 (15–50)	33 (14–51)	0.720**
Smoking status,* n (%)	Non-smoker	399 (48%)	399 (48%)	N/A
	Smoker	240 (29%)	240 (29%)	
	Ex-smoker	54 (7%)	54 (7%)	
	Passive smoker	20 (2%)	20 (2%)	
	Data missing	111 (13%)	111 (13%)	
Recorded rhinitis history,* n (%)		144 (17%)	144 (17%)	N/A
Oral corticosteroid courses/yr,* n (%)	0	709 (86%)	709 (86%)	N/A
	1	76 (9%)	76 (9%)	
	2	20 (2%)	20 (2%)	
	3–8	19 (2%)	19 (2%)	
Hospitalized for asthma,* n (%)		5 (0.6%)	5 (0.6%)	N/A
Socioeconomic quintile, n (%)	Lowest	309 (38%)	189 (23%)	<0.001 §
	2nd	49 (6%)	111 (13%)	
	3rd	297 (36%)	162 (20%)	
	4th	50 (6%)	167 (20%)	
	Highest	119 (14%)	192 (23%)	
SABA daily dose, † median (IQR)		0.55 (0–1.64)	0.82 (0.27–1.64)	<0.001**
Asthma consultations/yr	Median (IQR)	0 (0–1)	1 (0–2)	
	Range	0–9	0–14	
Asthma consultation rate, n (%)	0	524 (64%)	406 (49%)	<0.001**
	1	167 (20%)	212 (26%)	
	2	73 (9%)	103 (13%)	
	≥ 3	60 (7%)	103 (13%)	
Recorded gastroesophageal disease, n (%)		45 (5%)	62 (8%)	0.089 §
Recorded cardiovascular disease, n (%)		33 (4%)	48 (6%)	0.087 §
Daily dose of ICS on index day, ‡ median (IQR)		400 μg (400–800)	400 μg (400–800)	0.137**

IQR = interquartile range; N/A = not applicable (because matching criterion); SABA = short-acting β-agonist

*Matching criterion (number of oral corticosteroid courses and hospitalizations for asthma were during the year before the index date).

†One dose of SABA was defined as salbutamol 200 μg or terbutaline 500 μg.

‡Dose equivalents for beclomethasone dipropionate, budesonide, fluticasone propionate, BDP in solution (QVAR^®^, Teva UK), and mometasone were in a ratio of 1:1:2:2:2, respectively.

§χ^2 ^test, **Mann-Whitney

The median intended dose of ICS at the index date was similar for the two cohorts (see Table 3). Data for 601 of 824 patients (73%) in each cohort were used for these calculations; it was not possible to quantify the daily dose for the other 223 patients (27%) in each cohort because the instruction 'as directed' was recorded for their ICS prescriptions. Table 3 highlights that there were some differences (p < 0.05) between cohorts at baseline.

### Outcomes

**Figure 2:**
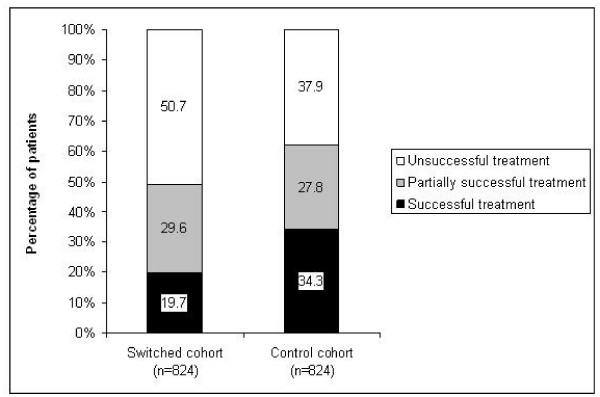
**Outcome of asthma treatment during study year 2 for patients whose inhaled corticosteroid device was switched without an accompanying consultation (switched cohort) and matched controls: percentages of patients experiencing successful treatment, partially successful treatment, and unsuccessful treatment (see text for definitions)**.

Fewer patients experienced successful treatment and more patients experienced unsuccessful treatment in the switched cohort than in the control cohort (Figure 2). The likelihood of successful treatment for the switched group was half that for the control group and fell to less than one third that for the control group after adjusting for confounding factors (Table 4). No other variables were predictive of success. Including the ICS dose in the model made no substantial difference: cohort membership remained the only predictor of success, with switched having significantly lower odds of success than control patients (OR, 0.30; 95% CI, 0.20 to 0.45; p < 0.001).

**Table 4:** Impact of ICS inhaler device switch: the likelihood of asthma control among switched vs. control patients during the year after the switch

	Unadjusted Analysis		Logistic Regression Analysis*	
	
	OR (95% CI)	p Value	OR (95% CI)	p Value
Successful treatment†	0.47 (0.37–0.58)	<0.001	0.29 (0.19–0.44)	<0.001
Unsuccessful treatment‡	1.69 (1.39–2.01)	<0.001	1.92 (1.47–2.56)	<0.001
Subsequent change in ICS device	1.73 (1.39–2.16)	<0.001	1.90 (1.50–2.42)	<0.001

CI = confidence interval; ICS = inhaled corticosteroid; OR = odds ratio

*Adjusted for baseline confounding factors, including short-acting β-agonist use, asthma consultation rate, socioeconomic status, and prescribed ICS dose at the index date (time of ICS device switch).

†Successful treatment vs. partially successful/unsuccessful treatment.

‡Unsuccessful treatment vs. successful/partially successful treatment.

The likelihood of unsuccessful treatment among switched patients was almost double that for control patients (see Table 4). Other variables predictive of unsuccessful treatment were a greater use of short-acting β-agonist and more asthma consultations at baseline. Including the ICS dose in the model made no substantial difference (OR, 1.93; 95% CI, 1.46 to 2.55; p < 0.001).

Patients in the switched cohort were also more likely than those in the control cohort to experience a subsequent change in ICS device or dose in year 2 (see Table 4). Other variables predictive of ICS change were short-acting β-agonist use, socioeconomic status, and rate of asthma consultation at baseline. Results were not substantially different when the ICS dose was included in the analysis (OR, 1.89; 95% CI, 1.48 to 2.40; p < 0.001). Subsequent changes in ICS device or dose during year 2 were recorded for 273 patients (33.1%) in the switched cohort and 183 patients (22.2%) in the control cohort (p < 0.001).

**Figure 3:**
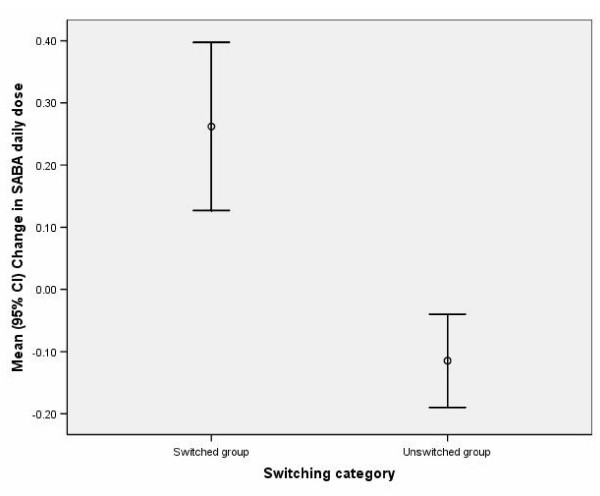
**Mean (95% confidence interval) change in daily short-acting β-agonist (SABA) dose from the baseline year (year 1) to the outcome year (year 2) for patients with switched inhaled corticosteroid device and unswitched individually matched controls**. One dose = salbutamol 200 μg or terbutaline 500 μg.

During year 2, the daily use of short-acting β-agonist increased in the switched cohort and decreased in the control cohort (Figure 3), and short-acting β-agonist use was greater in the switched than in the control cohort (Table 5). The switched cohort used 0.38 extra doses/day of short-acting β-agonist, compared with baseline use, than the control cohort (95% CI, 0.22 to 0.53; p < 0.001).

**Table 5:** Impact of ICS inhaler device switch: outcome measures for switched vs. control patients during the year after the switch

Outcome Measure		Switched Cohort (n = 824)	Control Cohort (n = 824)	p Value For Comparison
Oral corticosteroid courses/yr, n (%)	0	698 (85%)	721 (88%)	0.197†
	1	80 (10%)	65 (8%)	
	2	23 (3%)	12 (1%)	
	3–11	23 (3%)	26 (3%)	
Hospitalized for asthma, n (%)		3 (0.04%)	14 (1.70%)	NS
SABA daily dose,* median (IQR)		0.82 (0.27–2.19)	0.55 (0–1.64)	<0.001 §
Asthma consultation rate, n (%)	0	569 (69%)	514 (62%)	0.135†
	1	155 (19%)	177 (21%)	
	≥ 2	100 (12%)	133 (16%)	

ICS = inhaled corticosteroid; IQR = interquartile range; NS = not significant; SABA = short-acting β-agonist

*One dose of SABA was defined as salbutamol 200 μg or terbutaline 500 μg.

†χ^2 ^test, §Mann-Whitney

Most patients did not require a course of oral corticosteroids during the outcome year, and there were no significant differences between patient groups in the use of oral corticosteroids during this time (Table 5) or in the change in use from baseline. Similarly, there was no significant difference in asthma consultation rate between patients in switched and matched cohorts. Between year 1 and year 2, consultation rates fell slightly by a mean of 0.09 (SD, 1.33) in the switched cohort and by 0.35 (SD, 1.59) in the matched cohort. Thus, as compared with the baseline period, the switched cohort had 0.26 more asthma consultations in the outcome period than the matched cohort (95% CI, 0.11 to 0.40; p < 0.001).

There were very few hospitalizations for asthma during the study. Moreover, there was no significant difference between cohorts in admissions for possible asthma (Table 5) or in the change from baseline to outcome periods in admissions for possible asthma.

## Discussion

Switches in ICS device without clinical visit or consultation were associated with worsened asthma control in this retrospective analysis of a large UK general practice database. Patients whose ICS device was switched were significantly less likely to experience successful asthma treatment and significantly more likely to experience unsuccessful treatment than matched controls whose ICS device had not been switched. When possible baseline confounding factors were incorporated into the analysis, there was an even stronger association of device switch with lack of treatment success: patients with switched ICS device had less than one third the chance of successful treatment and were almost twice as likely to experience unsuccessful treatment or another change in ICS device compared with matched controls.

Use of short-acting β-agonist, a marker for asthma morbidity [16], increased significantly in the year after switching among patients in the switched cohort relative to controls. The number of hospitalizations for asthma and the number of oral corticosteroid prescriptions were, however, similar in the two cohorts during year 2.

We were able to identify over 100,000 individuals in the database who had undergone a switch in ICS device, and from these numbers were able to identify 55 practices where five or more patients had been switched in a 3-month period without a consultation, confirming our clinical impression that switching of ICS devices without a consultation is not an uncommon occurrence. While it is possible that some consultations occurred but were not recorded, given the multiple switching in these practices it is unlikely that the cases of switch without consultation are falsely identified. ICS device switching is typically implemented as a cost-saving measure. Indeed, in this study, the most common type of switch was from a dry powder inhaler to a metered-dose inhaler, often the cheapest inhaler device. Our study results would suggest, however, that the potential for worsened asthma control, and subsequent associated costs, may outweigh any acquisition cost savings from the switch.

Worsening asthma control after ICS device switching without a consultation could occur because of inadequate dosing secondary to poor technique or because of reduced patient adherence to therapy. Correct inhalation technique is necessary for attaining the full benefits of ICS.[9] However, inhaler devices are difficult to use, especially the metered-dose inhalers, which typically require the most patient instruction on their use [17,18]. Moreover, patient education and involvement in treatment decisions can improve adherence to therapy [18-20]; thus, it is possible that, without a clinical consultation, adherence was reduced in the switched cohort and asthma control worsened.

There are no other studies, to our knowledge, evaluating the switching of inhaler devices without a consultation. Surveys of the attitudes of health-care professionals and patients about the interchangeability of dry powder inhalers have consistently reported a generally negative attitude about switching dry powder inhalers without input from patient and physician [20-22].

This retrospective matched cohort study drew on data from a large clinical database and as such is limited by the completeness and accuracy of that database [23]. The GPRD is a very large primary care database that is managed with regular quality control and is considered to be valid for respiratory epidemiological research [15,24]. In theory, however, findings of statistical significance, but not necessarily clinical significance, can emerge from the evaluation of large data sets [23]. We limited our study population to 824 patients who could be individually matched to control patients using quite restrictive criteria. The parameters that were different at baseline for the two cohorts (short-acting β-agonist use, asthma consultations, and socioeconomic status) were included as factors in the regression model.

We chose a composite measure of asthma control to assess the impact of device switching because multiple outcome measures are the best means of determining effectiveness of asthma therapy [25]. The composite measures incorporated several measures of asthma morbidity, including daily short-acting β-agonist dose, change in asthma controller therapy, oral corticosteroid use, and hospitalization for asthma. Inclusion of lung function tests would have been impractical for this kind of study, as lung function tests are often not performed in primary care and are limited in interpretation by ceiling effects. The inclusion of OCS courses and hospitalizations means that a measure of severity was used to match patients, and surrogate measures of severity were entered into the regression model to reduce confounding.

There are a number of possible future studies given the findings of this analysis. The impact of age (child compared with adult) and long acting beta agonists are two possible sub-group analyses. However, care would need to be taken not to decrease the size of the cohort such that the power of the study becomes too weak to make conclusions from the findings.

## Conclusion

In conclusion, switching of ICS inhaler devices without a consultation is most commonly used for substitution of a cheaper metered-dose inhaler in place of a dry powder inhaler. Switching ICS inhaler devices without a consultation was associated with worsened asthma control. Therefore, the possible costs of adverse effects on asthma control should be weighed against savings in acquisition costs before instituting mass switching of ICS inhaler devices. Switching ICS inhaler devices without a face-to-face evaluation and consultation is inadvisable.

## Abbreviations

CI: confidence interval; COPD: chronic obstructive pulmonary disease; GPRD: General Practice Research Database; ICS: inhaled corticosteroid; IQR: interquartile range; OR: odds ratio; SABA: short-acting β-agonist; UK: United Kingdom.

## Competing interests

MT has received speaker honoraria from GlaxoSmithKline, Merck Sharp & Dohme, and Teva Pharmaceutical Industries and advisory panel honoraria from GlaxoSmithKline, Merck Sharp & Dohme, Teva, and Schering-Plough. He has received grants and research support for research in asthma from Asthma UK, GlaxoSmithKline, Merck Sharp & Dohme, and AstraZeneca.

DP has consultant arrangements with Altana Pharma, GlaxoSmithKline, and Ivax. He or his team have received grants and research support for research in asthma from the following organizations: UK National Health Service, Altana Pharma, AstraZeneca, GlaxoSmithKline, Ivax, Merck Sharpe & Dohme, Novartis, and Schering-Plough. He has spoken for Altana Pharma, GlaxoSmithKline, and Merck Sharpe & Dohme.

HC was at the School of Pharmacy, University of Bradford, Bradford, UK, at the time of this study. Prof. Chrystyn has been consultant to and received grants to attend conferences from the pharmaceutical industry, and he has received research grants and equipment from the pharmaceutical industry for his research group. The pharmaceutical companies are Aerogen, Altana Pharma, Boehringer Ingelheim, Eli Lilly, GlaxoSmithKline, Innovata Biomed, Meda Pharmaceuticals, Merck Sharp & Dohme, Omron, Orion, Ranbaxy, TEVA UK and Trinity-Chiesi. He has no personal shares with any pharmaceutical company.

AL, at the time of this study, worked for United BioSource Corporation (UBC) who were paid by GlaxoSmithKline for his time in this research project. UBC have worked with all of the major pharmaceutical companies to provide consultancy services. He has no personal shares and has never received any personal fees from any pharmaceutical company.

AW was an employee of GlaxoSmithKline R&D when this work was undertaken.

JZi is an employee of Respiratory Research, Ltd.

## Authors' contributions

MT, DP, HC, AL and AW all contributed to the conception, design and interpretation of the analysis of the study. MT, DP and JZ led the analysis of the dataset. All authors have been involved in the drafting of the manuscript and have given final approval of the version to be published.

## Pre-publication history

The pre-publication history for this paper can be accessed here:


